# Animal Models of Traumatic Brain Injury and Their Relevance in Clinical Settings

**DOI:** 10.1111/cns.70362

**Published:** 2025-04-16

**Authors:** Payal Chauhan, Nikita Yadav, Karan Wadhwa, Subbulakshmi Ganesan, Chakshu Walia, Gulshan Rathore, Govind Singh, Mosleh Mohammad Abomughaid, Abhilasha Ahlawat, Athanasios Alexiou, Marios Papadakis, Niraj Kumar Jha

**Affiliations:** ^1^ Department of Pharmaceutical Sciences Maharshi Dayanand University Rohtak India; ^2^ Department of Chemistry and Biochemistry School of Sciences, JAIN (Deemed to be University) Bangalore India; ^3^ Chandigarh Pharmacy College, Chandigarh Group of Colleges Jhanjheri Mohali India; ^4^ Department of Pharmaceutics NIMS Institute of Pharmacy, NIMS University Rajasthan Jaipur India; ^5^ Department of Medical Laboratory Sciences College of Applied Medical Sciences, University of Bisha Bisha Saudi Arabia; ^6^ University Centre for Research & Development, Chandigarh University Mohali India; ^7^ Department of Research & Development Funogen Athens Greece; ^8^ University Hospital Witten‐Herdecke Wuppertal Germany; ^9^ Department of Biotechnology & Bioengineering School of Biosciences & Technology, Galgotias University Greater Noida India; ^10^ Centre for Research Impact & Outcome, Chitkara University Institute of Engineering and Technology, Chitkara University Rajpura India; ^11^ School of Bioengineering & Biosciences, Lovely Professional University Phagwara India

**Keywords:** animal model, anxiety, clinical settings, trauma, traumatic brain injury

## Abstract

**Background:**

Traumatic brain injury (TBI) is a significant concern that often goes overlooked, resulting from various factors such as traffic accidents, violence, military services, and medical conditions. It is a major health issue affecting people of all age groups across the world, causing significant morbidity and mortality. TBI is a highly intricate disease process that causes both structural damage and functional deficits. These effects result from a combination of primary and secondary injury mechanisms. It is responsible for causing a range of negative effects, such as impairments in cognitive function, changes in social and behavioural patterns, difficulties with motor skills, feelings of anxiety, and symptoms of depression.

**Methods:**

TBI associated various animal models were reviewed in databases including PubMed, Web of Science, and Google scholar etc. The current study provides a comprehensive overview of commonly utilized animal models for TBI and examines their potential usefulness in a clinical context.

**Results:**

Despite the notable advancements in TBI outcomes over the past two decades, there remain challenges in evaluating, treating, and addressing the long‐term effects and prevention of this condition. Utilizing experimental animal models is crucial for gaining insight into the development and progression of TBI, as it allows us to examine the biochemical impacts of TBI on brain mechanisms.

**Conclusion:**

This exploration can assist scientists in unraveling the intricate mechanisms involved in TBI and ultimately contribute to the advancement of successful treatments and interventions aimed at enhancing outcomes for TBI patients.

## Graphical abstract

Traumatic brain injury (TBI) is a significant global health concern. Animal models are crucial for understanding the complex pathophysiology of TBI and developing effective treatments. This review explores common animal models of TBI, assessing their strengths and limitations in mimicking human injury. We discuss the relevance of these models in translating preclinical findings to clinical settings, paving the way for improved diagnosis, treatment, and management of TBI in humans.

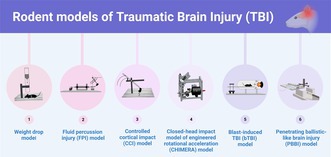

## 1 Introduction

A traumatic brain injury (TBI) refers to a significant injury to the brain that can cause temporary or permanent impairment of brain function. Undoubtedly, it plays a crucial role in causing disability and death in society, posing substantial challenges in terms of social, economic, and health aspects [1]. On a global scale, this issue is a significant health problem that leads to various health issues and disabilities. It impacts millions of people around the world each year, with an estimated annual occurrence of TBI ranging from 27 to 69 million cases worldwide [2, 3, 4]. In 2019, there was a considerable global health burden caused by TBI, with 27.16 million new cases, 48.99 million prevalent cases, and 7.08 million years lived with disability. From 1990 to 2019, there was a significant decrease of −5.5% in global age‐standardized incidence rates of TBI over nearly three decades. Regionally, Eastern Europe and high‐income North America carried the highest TBI burden in 2019. Nationally, Slovenia and Afghanistan reported the highest age‐standardized incidence rates of TBI, respectively [3, 5]. Approximately one million people in India suffer from serious brain injuries, with a yearly fatality rate of about 0.1 million [6]. TBI admission rates vary by age, with young children and the elderly being at higher risk [7, 8, 9]. According to data from the CDC and the National Institute of Neurological Disorders (NINDS), it has been found that falls are the leading cause of TBI [10].

In addition, the statistics on the Global Burden of Disease (GBD) in 2019 have further confirmed that falls are the primary cause of TBI in 74% of countries. Although falls are a major global cause, different countries and territories show variations in this regard [3]. Head injuries caused by objects or collisions, especially in sports or traffic incidents, are important factors in cases of TBI. In addition, TBI caused by domestic violence, shaken baby syndrome, or gunshot wounds to the head are classified as abuse‐related. These types of injuries can often result in physical, cognitive, and behavioral impairments, leading to temporary or permanent dysfunction [11, 12].

TBI is not just a single pathophysiological event, but rather a complex disease process. Following a TBI, people may face various difficulties that can be grouped into three main categories: physical challenges, cognitive impairments, and behavioral/emotional problems. Physical challenges may include issues with balance, muscle coordination, mobility, loss of sensory functions, bowel and swallowing difficulties, and more. Cognitive impairments can manifest as difficulties with attention, concentration, learning, planning, problem‐solving, speech, and reasoning. Behavioral and emotional problems may involve conduct disorders, attention issues, post‐traumatic stress, anxiety, depression, etc. [13, 14] There are two main types of TBI, one being primary brain injury. This type of injury occurs when the initial damaging forces cause tissue distortion and disintegration in the early post‐injury period. These initial forces can later trigger the creation of secondary damage [15].

In addition, various biochemical abnormalities that contribute to secondary injury have been identified. These include glutamate excitotoxicity, disturbances in cellular calcium balance, increased production of free radicals, lipid peroxidation, impaired mitochondrial function, inflammation, apoptosis, and diffuse axonal injury (DAI) [16, 17]. Secondary changes associated with both diffuse and focal TBI can result in neuronal death and neurological dysfunction. Like primary injuries, these secondary injuries have the potential to be reversed and can be affected by prompt and suitable treatment [18].

Anticipating symptoms of severe TBI can be quite difficult because of the wide range of complex outcomes that can occur with the brain [19]. However, as the individual recovers, the consequences tend to lessen. This process can extend over a period of weeks or even months. In the case of severe injuries, it may even take several years to notice specific changes [13, 14]. Severe TBI often results in more severe and potentially life‐threatening symptoms when compared to milder cases. Various factors, including the location and severity of the injury, the individual's age and overall health, and the quality of medical care received, can greatly impact the outcomes. TBI is a major contributor to injury‐related fatalities because of the unpredictable outcomes it can have. In addition, associations have been found between it and increased risks of neurodegenerative diseases, cognitive impairments, and personality changes [20, 21, 22]. Potential risk factors for TBI include pre‐existing psychiatric conditions, such as depression, substance use disorders, personality disorders, low socio‐economic status, being male, being young, and having previous head injuries [23, 24, 25, 26].

Several research studies also indicate that low educational attainment and depression have been found to be important risk factors linked to a higher likelihood of experiencing a TBI in the future. On the other hand, depression, nicotine dependence, and high aggression‐hostility are commonly observed outcomes following a previous TBI [27]. According to another study, it has been confirmed that around 40% of individuals who suffer from TBI have generally negative outcomes. Factors that can contribute to negative outcomes include delayed arrival time of more than 24 h, a low Glasgow Coma Scale (GCS) score, additional injuries, cerebrospinal fluid otorrhea, abnormal respiration, and hypoxia. Ensuring safe road traffic flow and enhancing healthcare services are crucial in mitigating the adverse effects of TBI in adults. Early intervention and comprehensive rehabilitation are crucial for improving outcomes in severe cases of TBI [28]. In addition, accurately predicting the symptoms of mild TBI (mTBI) poses challenges because of their diverse nature, which can be influenced by factors like the severity of the injury, age, overall health, and pre‐existing conditions [29, 30, 31]. After experiencing mTBI, individuals often exhibit a range of common symptoms, such as headache, confusion, dizziness, fatigue, blurred vision, memory problems, mood swings, sleep disruptions, nausea, ringing in the ears, and heightened sensory sensitivities. It is important to note that while many people make a full recovery from mTBIs, others may have lingering symptoms for weeks or even months. Seeking medical evaluation and treatment is crucial for proper management if a mTBI is suspected [32]. In a study conducted by Ponsford et al., the focus was on evaluating the progress of adults with mTBI within the first week and 3 months after the injury. The study also aimed to identify the factors that contribute to ongoing issues. The findings revealed that symptoms such as headaches, dizziness, and memory difficulties were commonly experienced by the 84 participants with TBI, as compared to the 53 individuals in the control group. At the 3‐month mark, the majority of symptoms had diminished, but a notable 24% of TBI participants continued to experience significant distress and disruption [33]. Factors associated with persistent symptoms included a history of head injury, neurological or psychiatric conditions, being a student, being female, and being involved in motor vehicle accidents. Many individuals in this group also exhibited signs of psychopathology. It is evident that various factors, apart from the severity of the injury, play a role in determining the outcomes of mTBI [33, 34].

There are several ways to assess the severity of TBI, and the most used assessment scales are the GCS score and the duration of level of consciousness (LOC) or post‐traumatic amnesia (PTA). These clinical evaluations assist in determining the extent of the trauma. Nevertheless, the degree of correlation between each of these severity assessments and outcomes remains somewhat uncertain. Every level of TBI can result in lasting physical, emotional, behavioral, and cognitive effects that have a permanent impact on a person's daily functioning and ability to resume work [35, 36]. TBI is classified by the GCS as mild, moderate, or severe. Around 75%–85% of traumatic brain injuries are categorized as mild TBIs, with a GCS score of 13–15 [37]. mTBI includes concussion, subconcussion, and certain blast injuries related to improvised explosive devices [38]. After a mild TBI people recover fully neurologically. However, a small percentage of individuals may experience long‐lasting changes in their neurocognitive and behavioral functions [36]. In the United States, it is estimated that 1.6–3.8 million concussions occur annually because of sport and recreation, with up to 50% of concussions going unreported [39]. Concussion is also quite common in collision sports such as football (American, Canadian, Australian), hockey (ice, field), boxing, martial arts, and wrestling with a considerable number of players at various levels being diagnosed with at least one concussion per season or even more severe brain damage [38, 40, 41, 42, 43]. Head injuries are a serious concern worldwide, often resulting from a variety of accidents. Traffic collisions, whether involving cars, motorcycles, bicycles, or pedestrians, can cause severe head trauma [44, 45]. Additionally, assaults and violence, including domestic violence and physical attacks, can result in traumatic head injuries [46, 47].

BOLD signal analysis, resting state functional connectivity, magnetic resonance imaging (MRI), and single‐photon emission computerized tomography (SPECT) imaging were previously used in the evaluation in identifying group‐level differences in mTBI patients [48, 49, 50, 51]. However, in recent years, structural and diffusion MRI sequences have gained prominence as clinically relevant tools for assessing mTBI [52, 53], enabling sensitive assessments of the brain's cellular structure [54, 55]. T1‐weighted imaging offers detailed anatomical views of the brain, while T2‐weighted imaging excels in detecting abnormalities like edema or inflammation [56, 57]. FLAIR imaging further aids in visualizing abnormal fluid within the brain [58, 59]. Diffusion tensor imaging (DTI) goes beyond measuring water diffusion patterns, providing valuable insights into the integrity of white matter tracts [60, 61, 62]. Studies have shown that DTI metrics can predict cognitive outcomes and correlate with the severity of injury [62, 63, 64, 65, 66, 67, 68]. Furthermore, functional MRI (fMRI) studies have also revealed notable changes in brain activation patterns following mTBI [69, 70, 71, 72, 73]. and these atypical brain activation patterns may endure for several months post‐injury. fMRI has also been employed to study changes in brain function following TBI [74]. Research has demonstrated that fMRI can detect alterations in brain activity patterns that correlate with cognitive deficits. It also has been used to investigate the effects of TBI on brain function and to evaluate the efficacy of potential therapeutic interventions [75, 76, 77]. Patients with moderate TBI (GCS 9–13) may experience initial lethargy or stupor, while those with severe TBI (GCS 3–8) may be in a comatose state and unable to open their eyes or respond to commands. Severe TBI patients face a significant risk of experiencing secondary brain injury, which may involve issues such as low blood pressure, low oxygen levels, and swelling of the brain. Lower GCS scores [3, 4, 5, 6, 7, 8, 9], typically seen in cases of severe TBI, are strongly linked to unfavorable outcomes such as severe neurological disability, vegetative state, and death. Advancing age, especially over 60 years old, is also associated with a higher risk of a negative outcome [78].

Despite progress in preclinical research for TBI, clinical trials often fail due to the variability of TBI cases and limited diagnostic tools. Current diagnostic methods, like the GCS and neuroimaging, can be subjective and unreliable. Researchers suggest that analyzing biomarkers in blood after TBI could provide a more accurate way to classify injuries, predict complications, and evaluate the effectiveness of treatments [79, 80]. Using biomarkers alongside traditional methods could improve the design and outcomes of clinical trials. By tracking biomarker changes over time, doctors can better monitor patient status and tailor treatment plans. Stratifying patients based on biomarker profiles can enhance patient safety and improve the accuracy of trial results. Additionally, analyzing patient groups based on specific outcomes can help identify subgroups that benefit most from treatment and potential side effects [81]. Several biomarkers are associated with neuronal injury, including ubiquitin carboxy‐terminal hydrolase L1 (UCH‐L1), microtubule‐associated protein 2 (MAP2), neuron‐specific enolase (NSE), neurofilament, and glial fibrillary acidic protein (GFAP) [82, 83, 84].

Neurofilament light chain (NFL), a marker of axonal injury, has been shown in both rodent and human studies to correlate with the severity of TBI. Serum NFL shows promise as a biomarker for acute and repetitive sports‐related concussions and for patients with sub‐acute and chronic TBI. It was found that the level of phosphorylated NFL‐H (pNFL‐H) has been shown to increase in amateur boxers after a match [85]. This biomarker has also been linked to the severity of mTBI in studies. Researchers have found that higher pNFL‐H levels in blood samples correlate with CT scan findings, suggesting their potential usefulness in determining which individuals need further imaging to assess injury extent [86, 87]. Similar findings have been observed with neurofilament heavy chains (NFH). Elevated NFH levels have been associated with DAI in both children and adults with TBIs. These studies highlight the potential of neurofilament proteins as biomarkers for TBI severity and prognosis [88, 89]. Elevated NFL levels have been associated with worse outcomes, suggesting its potential as a prognostic biomarker [90]. In a study, early levels of NFL in patients with mTBI may predict the severity of DAI, a type of brain damage, in the weeks and months following the injury [81].

GFAP is also a promising biomarker for brain injury screening. Research has shown that the levels of GFAP or its breakdown products, GFAP‐BDP, increase in both CSF and blood serum following mild, moderate, or severe TBI in adults. These elevated GFAP levels are correlated with the severity of the TBI and patient outcomes [91]. As an indicator of astrocytic injury, GFAP levels increase in blood following TBI in both rodents and humans. Research suggests its utility in predicting outcomes and monitoring the progression of brain injury [92]. Additionally, research suggests that GFAP may be a brain‐specific marker for malignant gliomas. Some studies have indicated that measuring both GFAP and UCH‐L levels in the blood could help differentiate between focal and diffuse injuries in severe TBI patients [93, 94]. Serum GFAP levels rise in the initial days following a severe TBI and are associated with clinical outcomes. A recent study found a strong correlation between plasma levels of GFAP and UCH‐L1 and the GCS, suggesting their potential for acute phase diagnosis of TBI within the first 2 days after injury. Several studies have also demonstrated a link between GFAP levels and increased intracranial pressure. For instance, Pelinka et al. found significantly elevated GFAP serum concentrations in patients with ICP greater than 25 mmHg [94, 95]. Recent research by Papa et al. focused on the time course and diagnostic accuracy of GFAP and UCH‐L1 in patients with and without mTBI. They found that GFAP consistently detected mild to moderate TBI, predicted CT lesions, and neurosurgical intervention over 7 days, while UCH‐L1 acted as an early marker [96].

S100B has also been identified as a biomarker for blood‐brain barrier (BBB) damage. It has also been suggested as a potential predictor of outcomes following TBI. Studies have shown that combining serum levels of S100B with those of NSE and GFAP can improve the predictive value for TBI outcomes [97, 98]. S100B levels have been shown to correlate with the severity of brain injury. Numerous studies have reported associations between S100B levels and clinical outcomes such as the GCS and mortality. There is also extensive literature discussing the potential of S100B as a prognostic biomarker in serum or urine for severe TBI [99, 100]. Studies suggest a correlation between S100B levels in both serum and CSF and ICP, indicating its potential for detecting intracranial hypertension. Significant associations have been found between elevated ICP values and peak S100B concentrations in both serum and CSF 135–137 [101, 102]. In patients with severe TBI, both CSF and serum S100B concentrations were positively correlated with increased ICP and progressive intracranial hemorrhage. Further research has shown that S100B levels are correlated with increased ICP in both the early and late phases following TBI. In contrast, another biomarker, NSE, was only weakly associated with ICP elevation in the late phase, suggesting that S100B is a more accurate predictor of increased ICP [103].

Recently, animal models have played a vital supporting role in underlining the cellular and molecular pathogenesis of TBI [104]. Several different species, such as cats, dogs, and non‐human primates, have been used to study the underlying causes, genetic connections, long‐term effects, and experimental models in brain injury research. Nevertheless, rodent TBI models have become the preferred option in laboratory research. This is because they allow for easy surgery, provide reliable statistical data analysis, can accurately detect differences between groups, and are more cost‐effective in terms of purchasing, handling, and lodging animals [31, 105].

In addition, rodent models are essential in TBI research because they can accurately replicate various injury mechanisms, including penetrating injuries, contusions, and concussions that have clinical manifestations. These models provide a valuable platform for studying injuries of different degrees, ranging from minor to severe traumas. Monitoring these animals requires neurobehavioral measurements and neuropathological assessments, which are crucial performance criteria. Despite the intricate nature of the subject, preclinical studies on potential treatments for TBI have not yet been effectively translated into clinical trials, despite showing promising results. Animal models of TBI are designed to produce a consistent type of injury. Therefore, it is important to note that animal models may not completely mimic all aspects of secondary injury development seen in human TBI [16, 17, 106].

Understanding the basic injury mechanisms of TBI requires testing experimental therapies on animals first to ensure their safety and effectiveness before moving on to human trials. This comprehensive review aims to critically evaluate the current state of animal models in TBI research. By addressing the limitations, challenges, and clinical relevance of these preclinical models, it seeks to provide a roadmap for improving their translational potential. Specifically, the review will delve into the shortcomings of existing animal models, emphasizing the need for more human‐relevant endpoints and biomarkers. Furthermore, it will explore the complex interplay of biological, psychological, and environmental factors that influence TBI outcomes, underscoring the importance of a nuanced approach to preclinical research. By critically analyzing the strengths and weaknesses of current animal models and research strategies, the review aimed to guide future studies toward the development of more clinically relevant and effective therapeutic interventions for TBI.

## 2 Pathophysiological Changes Associated With TBI

TBI is recognized as an intricate neurological disorder that involves social disruption and cognitive dysfunction. Immediate TBI can cause substantial alterations at the tissue and cellular levels, resulting in enduring neurological deficits [107]. It is an intricate neurological condition that occurs when there is rapid movement within the skull, leading to mild to moderate injuries. These injuries can cause symptoms like nausea, headaches, fainting, and forgetfulness. The time it takes for these symptoms to resolve can vary from a few days to several weeks, depending on the severity of the injury [107, 108]. Undoubtedly, TBI is a significant global factor in causing illness and death [109]. Brain injuries have unfortunately become a leading cause of death among teenagers and young individuals. Every year, around 69 million people are affected by TBI, with vehicle traffic accidents being the leading cause [2, 110, 111].

**FIGURE 1:**
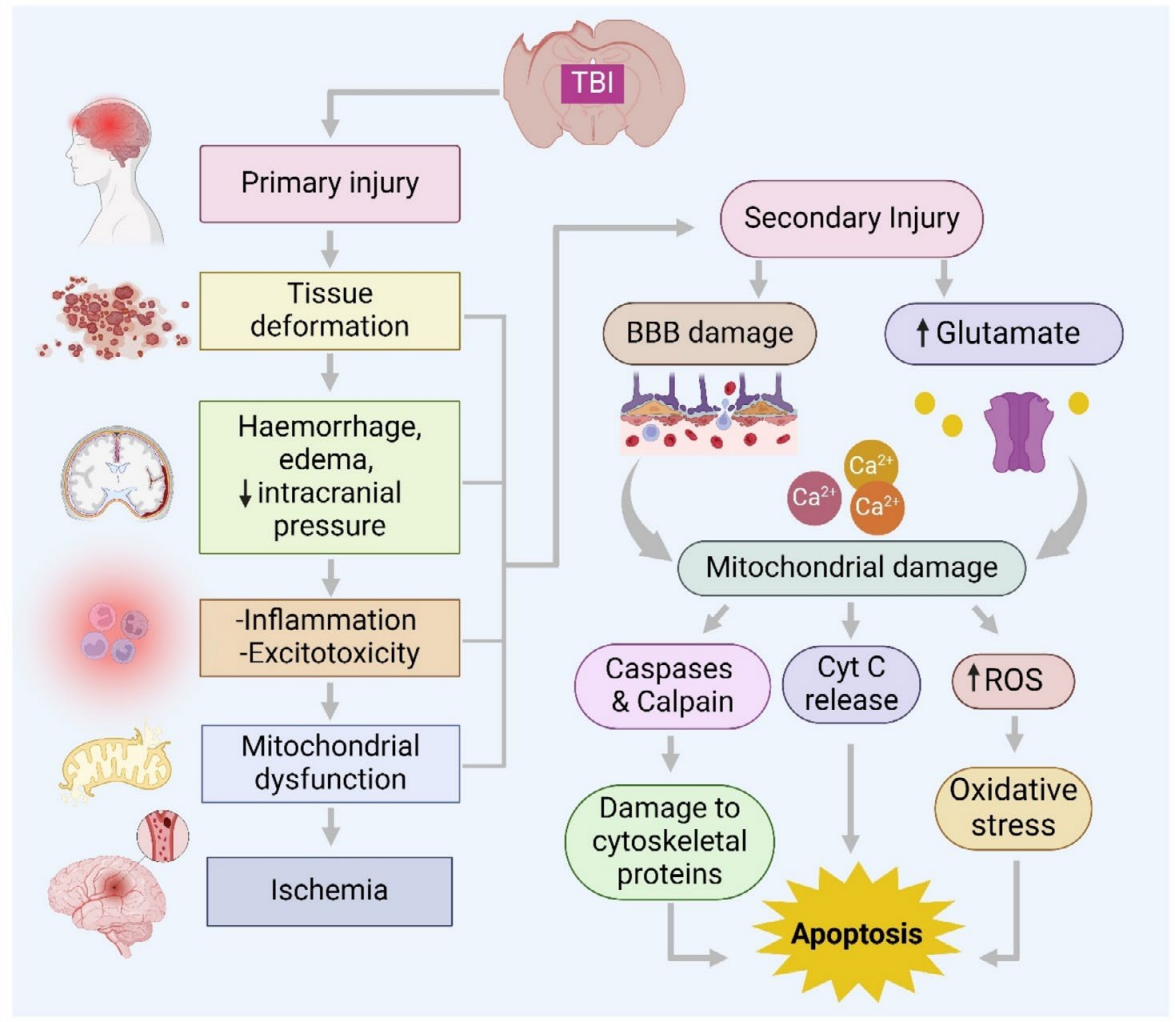
Pathophysiological cascade associated with TBI.

TBI is divided into two main categories: open head injury (OHI) and closed head injury (CHI). When it comes to CHI, the initial impact sets off a chain reaction of tissue damage, which in turn triggers additional injury or biochemical cascades that worsen the damage [1]. Concussion and sub‐concussion are primarily caused by the rapid acceleration and deceleration forces that affect the brain, either rotationally or linearly. When external forces affect the head, the brain experiences deformation and elongation, resulting in the stretching of its components like glial cells, neurons, and blood vessels. Stretching can have an impact on the functioning of brain components such as axons, glial cells, and dendrites [112]. During the early stages of brain damage following a TBI, there are issues with regulating cerebral blood flow, abnormal metabolism, and direct tissue damage. This pattern of ischemia results in the buildup of lactic acid due to anaerobic glycolysis, which leads to the development of edema and an increase in membrane permeability [113], as illustrated in Figure 1. After a few days, the secondary injury phase kicks in. This phase is reversible and involves changes in ionic balance, release of neurotransmitters, dysfunction of mitochondria, death of neurons, degradation of lipids, and the start of inflammatory and immune responses [1, 12].

Initially in the secondary injury phase, the modulation in the several neurotransmitters release, such as elevation in extracellular glutamate, reduced gamma‐aminobutyric acid (GABA) receptor binding [114], dysregulated acetylcholine (ACh) release [115], increased nor‐epinephrine levels [116], the unregulated release of dopamine [117], and decreased serotonin transportation [118], can trigger a cascade of events that subsequently induces apoptotic neuronal loss. During this stage, there is a significant increase in the release of certain neurotransmitters, like glutamate and aspartate. This happens because the cell membrane becomes depolarized, which then activates various receptors and ion channels, such as N‐methyl‐D‐aspartate (NMDA) receptors, amino‐3‐hydroxy‐5‐methyl‐4‐isoxazolpropionic acid (AMPA) receptors, and voltage‐dependent Na^+^ and Ca^2+^ ion channels. When Na^+^ and Ca^2+^ ions enter neuronal cells, it sets off intracellular processes that involve self‐digestion. After the activation of certain enzymes, such as phospholipase, lipid peroxidase, and protease, there is an increase in the levels of free fatty acids and free radicals [119, 120, 121, 122].

As a result, the increased Ca^2+^ influx leads to a breakdown in anaerobic metabolism, which in turn disrupts cellular energy levels. This causes a decrease in ATP storage and the malfunctioning of energy‐dependent membrane ion pumps. In the second stage, there is a depolarization of the terminal membrane, activation of NMDA, and an excessive release of excitatory neurotransmitters. This leads to dysfunction in membrane ion pumps. Furthermore, the activation of endonucleases, caspases, and translocases triggers changes in biological membranes and nucleosomal DNA, resulting in programmed cell death (apoptosis) or necrosis [120, 121]. There is a notable connection between mitochondrial dysfunction, glutamate excitotoxicity, blood–brain barrier (BBB) disruption, and cognitive impairment as well as neuronal cell death in TBI. Through scientific experimentation, a deficiency in mitochondrial bioenergetics has been observed to persist for a duration of up to 14 days following a TBI. Scientific studies have shown that when glutamate‐induced excitotoxicity occurs, it can cause damage to the structure and function of mitochondria. This damage is caused by an increase in intracellular Ca^2+^ and an overload of mitochondrial Ca^2+^, which in turn leads to an increase in mitochondrial permeability [106, 123]. This process results in the release of cytochrome C into the cytoplasm, which triggers apoptosis by binding with apoptosis‐activating protein‐1 and causing oxidative stress through ROS. Damage of this nature plays a role in triggering the cell death pathway, hindering synaptic plasticity, and causing axonal injury. These factors ultimately result in cognitive deficits following a TBI [124]. When NMDAR is activated by glutamate, it produces reactive oxygen species (ROS) and reactive nitrogen species (RNS), resulting in the formation of a highly reactive species called peroxynitrite. This peroxynitrite can be harmful to neuronal cells. Cell death in TBI can manifest in two different ways: necrosis and apoptosis. Apoptosis is a form of cell death that occurs as part of a programmed process, whereas necrosis is the outcome of tissue damage caused by lack of oxygen or physical trauma. This damage leads to the excessive release of excitatory amino acid neurotransmitters and metabolic failure [125].

Indeed, TBI can be caused by a range of factors, such as road accidents and sports injuries. These incidents can result in inflammation, mitochondrial damage, and ion imbalance. Animal models have greatly contributed to the extensive research on TBI. These models allow researchers to recreate injuries at the molecular, cellular, and organismal levels, offering valuable insights into the mechanisms and effects of TBI. Through replicating the intricacies of TBI in controlled environments, animal models play a crucial role in enhancing our comprehension of the underlying mechanisms and provide a valuable platform for evaluating potential therapeutic interventions.

## 3 Animal Model of TBI

Animal models are essential for conducting thorough comparisons of different human conditions. By adopting a scientific approach, researchers can gain a deep understanding of how diseases progress. This knowledge empowers them to develop treatment protocols that can be fine‐tuned for maximum effectiveness, even before conducting human testing. Various models have been developed to study a range of brain‐related conditions, including TBI [126, 127]. Animal models of TBI have been instrumental in advancing our understanding of potential treatments that can mitigate oxidative stress, improve BBB permeability, and address biochemical impairments associated with TBI [126, 128]. Behavioral deficits associated with human TBI lead to disruptions in information processing at different levels. Memory, information‐processing speed, and efficiency are among the cognitive domains that are most impacted [15].

Research has shown that TBI can cause lasting effects on memory, specifically affecting both retrograde and anterograde memory. These memory abnormalities tend to have a greater impact on long‐term memory processes compared to other cognitive functions [10, 129]. Research has shown that head injuries can have an impact on memory function [129]. Long‐term memory processes are significantly impacted after a TBI. Experimental models for TBI aim to replicate the clinical features of the condition, including motor, neurobehavioral, and cognitive impairment observed in humans [130]. When studying TBI animal models, it is important to ensure that the extent of tissue damage aligns with the injury degree. This can be determined by observing changes in fixed parameters. As an example, in the weight drop model, adjusting the falling height has an impact on the severity of the injury [131, 132].

Most TBI models are typically carried out on animals, particularly rodents. Although these models offer valuable insights, there are inherent variations in brain structure, function, and response to injury among different species [30]. Several animals, including rodents, sheep, pigs, dogs, monkeys, goats, drosophila, and zebrafish, are commonly used as animal models for studying TBI [133, 134]. Utilizing mouse models for TBI offers numerous benefits, such as cost‐effective breeding, ease of handling, and the opportunity to generate genetically engineered variants through innovative technologies, as shown in Table 1. Using knockout and transgenic mice, researchers have been able to gain valuable insights into the underlying causes of TBI by studying the effects of functional molecule loss or gain.

**TABLE 1:** SWOT analysis of different animal models of TBI.

TBI model	Strength	Weakness	Opportunities	Threats
Weight drop model	Produces a scalable TBI by varying the drop height and weight mass The device construction is simple, and the kinetic mechanisms are easy to understand [135] Induces DAI to a greater extent with significant damage to microvasculature and BBB [132, 136]	It takes strong forces to harm the brain through an unbroken skull A plate needs to be inserted at the impact location to redistribute the force of the damage to lower the high incidence of skull fractures [10, 137]	Widely used to mimic DAI without focal lesions [138]	High mortality rate Indirect forces that affect the brain through bone cause a greater range of brain injuries [10, 137]
FPI model	Highly reproducible with fine‐tuning Direct brain injury without skull protection Produces scalable injuries [139] Causes significant brain damage with DAI‐like symptoms [10]	Require craniotomy Care must be taken when setting up this system because mistakes like improper hub installation and residual air in the system can result in a variety of injuries, including dural adhesions and tears [28, 139]	Mimic various clinical features of human TBI including deformation of the brain, brain concussion, and contusion, including sports‐related TBI [140]	High mortality [141, 142]
CCI model	Low mortality and simple preparation [143] Cause reproducible focal brain damage [144]	Equipment is costly [145] DAI or severe scale neurological or functional deficits are not caused by small diameter impactor tips High frequency of dural tears, which makes it difficult to reproduce closed TBI [10, 146] Require craniotomy [146, 147, 148]	Mimics human close head injury, including contusions [149]	Triggers several complications including hemorrhage and ischemia [10, 119]
CHIMERA model	Completely nonsurgical Enables exact mechanical input control and reproducible head kinematics [10, 132]	Expensive equipment [145] Need further characterization [150, 151]	Replicates several key behavioral, biochemical, and neuropathological characteristics of human TBI including axonal injury, neuroinflammation, and functional deficits [127]	Can only be used for diffused injury, not for focal injury [127]
ACHI model	Do not require craniotomy [152, 153] No mortality [154] Results in mild injuries without fatalities, skull fractures, or brain bleeds [144, 155]	Need standardization [30]	The injury exhibited similarities to the clinical counterpart [30, 154]	Can only be used for diffused injury, not for focal injury [30]
bTBI model	Injury mechanism close to military TBI [120, 156] Minimizes the ocular and head acceleration blast effects [10]	Need standardization [157, 158]	Mimics transient vestibulomotor deficits and persistent orofacial pain with significant behavioral and cognitive impairment [156, 159]	Due to lung injury from intense high‐pressure shock waves, the animal may die [160] The neurovascular unit is negatively affected by TBI caused by a blast‐induced shock wave which raises the risk for AD [161]
Acceleration‐deceleration–induced TBI model	Reproducible model [132]	Need standardization [162] Model is technically sophisticated and expensive	Injury mechanism close to human TBI [163]	Duration of coma is associated with the extent of DAI [164]
PBBI model	Simple and easy to control [30]	Need standardization [10, 119]	Effective in investigating BBB permeability, brain swelling, cognitive function, and motor impairment [10, 119]	Does not differentiate all clinical phases of tissue injuries [165] Can only be used for focal injury [10, 119]

Three different types of models can be used to categorize TBI: focal, diffuse, and non‐impact injury. These models closely resemble the clinical presentations of TBI. Every model follows precise procedures and produces specific results, intending to offer a valuable understanding of the various scenarios in which head trauma can occur in humans [15, 127, 128]. In addition, these models offer the flexibility to modify injury severity levels, which helps in gaining a more comprehensive understanding of how injuries progress. By conducting these experiments, one can make comparisons between different levels of human injury severity, which can ultimately contribute to the development of improved diagnostics and treatment protocols.

### 3.1 Animal Model of TBI With Direct Head Impact

#### 3.1.1 Weight Drop Model

**FIGURE 2:**
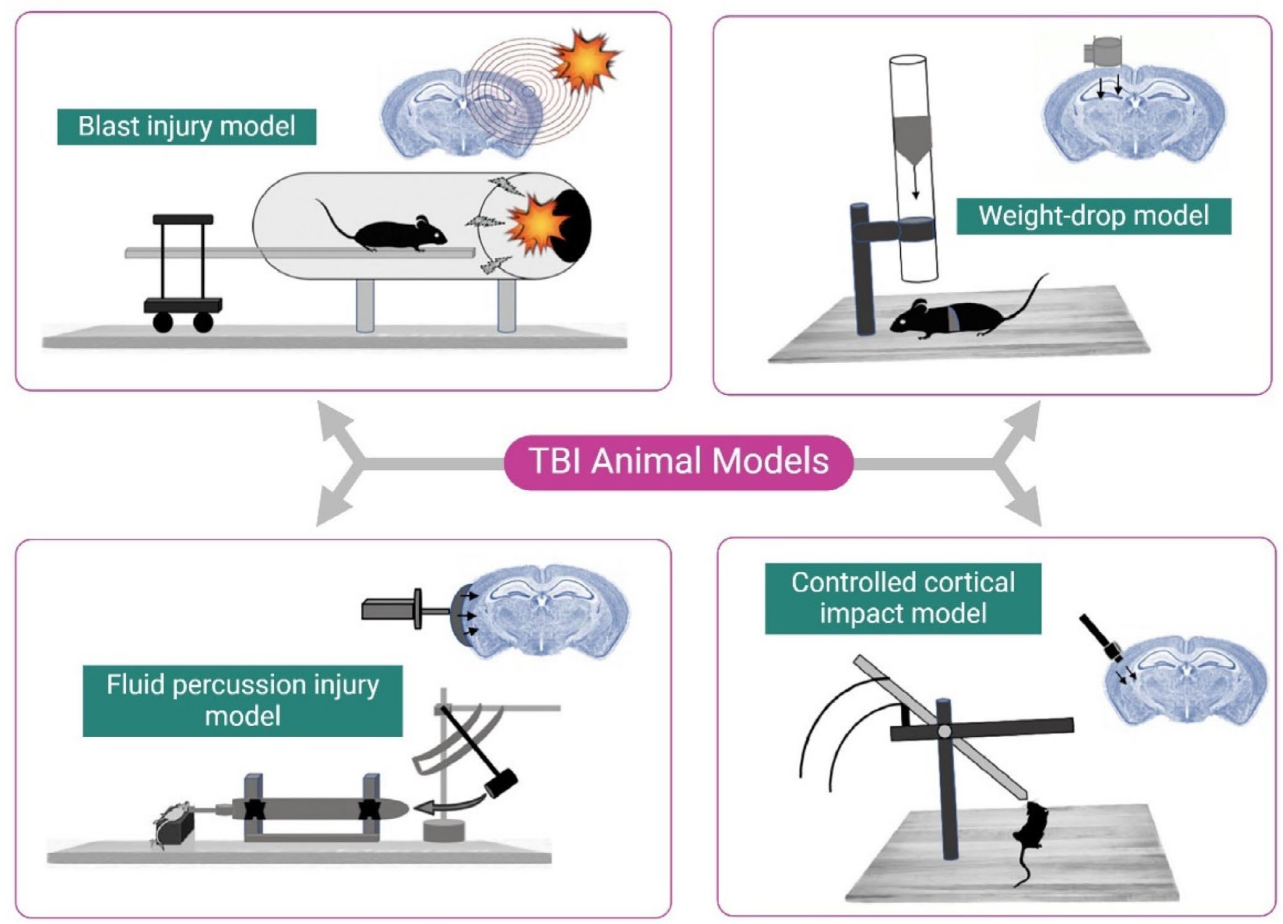
Overview of various animal models of TBI.

Weight drop models have been used in rodents for many years to improve our understanding of the pathophysiology of TBI. Researchers have adapted the weight drop paradigm for use in mice after initially developing it for rats [10, 119]. In 1994, Marmarou and colleagues developed a groundbreaking closed‐skull TBI model in rats. They achieved this by utilizing a weight drop method [166]. The impact acceleration model developed by Marmarou aims to replicate cases of TBI in humans caused by falls or motor vehicle accidents [28, 167, 168]. It was designed to facilitate the study of both focal and diffuse brain injuries in rats and mice. This particular model differs from other TBI models that result in focal brain contusion, as it instead produces shear stress and DAI. The process entails carefully shaving the scalps of anesthetized mice, creating an incision to reveal the periosteum, and securely affixing a stainless steel helmet to the skull using dental acrylic. Through careful design, the helmet effectively distributes kinetic energy across the brain, minimizing the risk of concentrated injury. An array of brass weights descends through a plexiglass column, spanning from 50 to 500 g. This controlled experiment consistently produces an injury when released from a predetermined height onto a rat positioned on a foam bed with a documented spring constant, as depicted in Figure 2.

In the initial model, it was observed that there was a mortality rate of 44% and a skull fracture rate of 12.5% when a weight of 450 g was dropped from a height of 2 m. Seizures, apnea, and hypertension were observed alongside the injury, and it was found that the use of mechanical ventilation improved survival rates. Upon examination after death, the analysis showed swelling in the brain, enlargement of the ventricles, and significant harm to neurons, axons, and microvasculature. It particularly affects important areas such as the corpus callosum, internal capsule, optic tracts, cerebral and cerebellar peduncles, and the long tracts in the brainstem [169]. This model also elicits motor and cognitive deficits, including challenges in beam walking and memory [30, 119, 169, 170, 171]., similar to those observed in cases of other direct head impact trauma models including fluid percussion injury (FPI) and controlled cortical impact (CCI) models. These deficits exhibit a correlation with the severity of the injury [172, 173]. Although weight‐drop models have a drawback of higher variability in injury severity, they provide a cost‐effective and easy‐to‐implement method that can produce graded DAI similar to what is observed in human TBI. Therefore, the transmission of acceleration, deceleration, and rotational forces to the brain results in the production of clinically significant symptoms [174]. Various researchers have made adjustments to this model to explore different clinical and behavioral outcomes. These modifications include using a stereotactic frame, foam cushion, or resin mold to support the head. Various factors have been standardized, such as the type of head support, mass weight, and height of its fall. Additionally, the severity of brain damage can be influenced by the level of calcification and thickness of the skull. However, researchers have found that changes in the angle of impact can also affect the nature of brain injuries and the resulting skull fractures [119, 175]. Shapira and colleagues have devised a novel rat weight‐drop model that utilizes a free‐falling rod in place of traditional weights. This variation leads to BBB disruption, cerebral edema, neurological deficits, focal contusion, and cell death [131]. In Feeney's weight drop model, craniotomy was performed to deliver the weight to the intact dura, which led to cortical contusion. From a scientific perspective, these injuries develop as hemorrhages in the white matter beneath the contused cortex within the first few hours after the injury occurs [136]. Adelson et al. also made modifications to Marmarou's model to induce diffuse cortical swelling. Recognizing the significant mortality rate linked to the 450‐g weight, they researched the effectiveness of a weight ranging from 75 to 125 g dropped from a height of 2 m. The study found that dropping a 100‐g object from a height of 2 m caused a fatal head injury, but there was no evidence of brainstem hemorrhage or disruption. Additionally, there was a notable decrease in mortality during the immediate aftermath of the injury [176].

A recent study has confirmed that dropping a 10‐g weight from a height of 10 cm onto the exposed right brain of rats leads to the formation of a glial scar and is linked to cognitive impairments [177]. A study conducted by Khalin et al. in 2016 discovered that when a weight of 250 g was dropped from a height of 2 cm, it resulted in a significant closed head injury in male C57BL/6 mice [178]. Interestingly, the weight drop model for rodents has also been adapted for use in adult zebrafish. Maheras and his team conducted an experiment where they induced diffusive injury in the brain of adult zebrafish. This was achieved by striking a 0.33‐g ball bearing, which delivered a force of 0.0032 N and an impact energy of 35 mJ. A study conducted on adult zebrafish used RNA sequencing analysis to examine gene expression at two different time points after injury. The analysis identified genes that showed significant differences in expression and were associated with pathways related to injury response, central nervous system injury, and neurodegeneration [179]. Multiple studies have utilized the weight drop model to demonstrate its clinical relevance, as outlined in Table 2. Multiple studies have shown that when rodents are exposed to the weight drop model, they experience neuronal death, cognitive deficits in different behavioral settings, an increase in apoptotic markers, and signs of neuroinflammation. These findings collectively suggest the presence of a closed head injury [135, 137, 177, 182, 212, 213]. Machado et al. found that the levels of PC1 (cytokines and chemokines) differed between the two hemispheres, while the expression of PC2 (neurotrophic factors) only varied across the specific brain regions they studied. The model developed in their study effectively replicates the clinical signs and pathological features commonly observed in cases of mTBI. This model shows great potential as a valuable tool in furthering our comprehension of the pathophysiology of mTBI and in evaluating potential therapeutic targets [180]. However, the results of these studies indicate that using weight drop injuries in animal models can be useful for further testing of neuroprotectants for the brain. These findings may have implications for understanding TBI in humans.

**TABLE 2:** Several preclinical investigations utilizing rodent models of TBI and their correlation with clinical pathogenesis.

Model	Type of head trauma/injury	Model organism	Findings correlated with clinical significance	Days	References
Weight drop model	Direct/Diffused	Male C57BL/6 mice	↑ Cognitive impairments	7 days	[178]
	Direct/Diffused	Sprague–Dawley rats	↑ Glial scar formation ↑ Cognitive impairments	14 days	[177]
	Direct/Diffused	Male C57BL/6 mice	↑ Cytokines and oxidative stress level ↑ Locomotor impairment ↓ Spatial memory ↑ BBB disruption	6 h, 24 h, 72 h, and 30 days	[180]
	Direct/Diffused	Male C57BL/6 mice	↑ Neurodegeneration ↑ Microglia level	7 days	[181]
	Direct/Diffused	C57BL/6 male mice	↑ A*β* deposition ↑ Acute cognitive impairments	28 days	[182]
FPI model	Direct/Mixed	Male Long‐Evans rats	↑ Cognitive impairment ↑ Neuroinflammation	24 h and 8 weeks	[183]
	Direct/Mixed	Male Long‐Evans rats	↑ Neuroinflammation	24 h and 4 weeks	[184, 185]
	Direct/Mixed	Juvenile C57BL/6J mice	↓ Cognition ↑ Level of inflammatory cytokines ↑ Microglia accumulation	48 h	[186]
	Direct/Mixed	Male Sprague–Dawley rats	↑ Seizures ↑ Expression of CB1 receptor and 2‐arachidonoylglycerol (2‐AG)	12 months	[187]
	Direct/Mixed	Sprague–Dawley rats	↑ Seizures ↑ Intestinal permeability and endotoxemia	7 months	[188]
	Direct/Mixed	Sprague–Dawley rats	↑ Seizures	7 months	[189]
	Direct/Mixed	Sprague–Dawley rats	↑ Seizures	7 months	[190]
CCI model	Direct/Focal	Sprague–Dawley rats	Alter Ach transmission ↑ Structural damage and neurological alterations	2 weeks to 12 months	[191]
	Direct/Focal	Male C57BL/6J mice	↑ Neuronal degradation ↑ DAI ↑ Motor deficits	3 days	[117]
	Direct/Focal	Male C57BL/6J mice	↓ Cognition ↑ Neuronal damage	28 days	[192]
	Direct/Focal	Sprague–Dawley rats	↑ Neocortical hyperexcitability ↑ Spontaneous epileptiform firing	16 days	[193]
	Direct/Focal	C57BL/6 mice	↑ Incidence of spontaneous seizures	5 months	[194]
	Direct/Focal	C57BL/6 mice	↑ Seizure response	3 months	[195]
	Direct/Focal	Male C57BL/6 mice	↑ Spontaneous recurrent seizures ↑ Contralateral hippocampal sclerosis	4 months	[196]
	Direct/Focal	Male Wistar rats	↑ Epileptogenesis ↑ Expression of TNF‐α, TGF‐β, IL‐10	48 h	[197]
	Direct/Focal	Male Wistar rats	↑ Chemical and electrical kindling	7 days	[198]
CHIMERA	Direct/Diffused	Male C57BL/6 mice	↑ DAI ↓ Spontaneous behavior	Up to 30 days	[199]
	Direct/Diffused	C57BL/6 mice	↑ Neuroinflammation and BBB leakage ↑ Micro structural vascular abnormalities, and gray matter microgliosis	60 days	[200]
ACHI	Direct/Diffused	Male Long‐Evans rats	↑ Sensorimotor deficits ↓ Spatial memory ↑ Level of neurofilament light	7 days to 3.5 months	[29]
	Direct/Diffused	Male Long‐Evans rats	↑ Neuroinflammation ↑ Neurobehavioral deficits	14 days	[201]
	Direct/Diffused	Juvenile Long Evans rats	↑ Memory and behavioral impairment	7 days	[155]
	Direct/Diffused	Juvenile rats	Alter synaptic plasticity	7 days	[202]
bTBI model	Indirect/Diffused	Wistar Rats	↑ Memory loss ↑ Intracranial pressure	7 days	[203]
	Indirect/Diffused	Male ICR mice	↑ Neuroinflammation ↓ Cognitive ability ↑ BBB damage	7 days and 30 days	[156]
	Indirect/Diffused	Sprague–Dawley rats	Produce primary injury ↑ Behavioral changes ↓ Cognition	4 days and 30 days	[204]
	Indirect/Diffused	Male C57BL/6J mice	↑ Axonal injury ↑ Neurological deficits ↓ Memory ↑ Mitochondrial abnormalities and myelin deficits	7 days and 30 days	[205]
	Indirect/Diffused	Male Sprague–Dawley rats	↓ Behavioral and cognitive function ↑ Neuronal injury	48 h	[206]
Acceleration–deceleration‐induced TBI model	Indirect/Diffused	Sprague–Dawley rats	↑ Tau and neurofilament heavy chain ↑ APP and axonal injury ↓ Behavior functions	3 days, 7 days and 14 days	[207]
	Indirect/Diffused	C57/BL6 mice	↓ Cerebral blood perfusion ↑ BBB permeability and brain edema ↑ Neuroinflammation	3 to 30 days	[208]
PBBI model	Indirect/Focal	Sprague–Dawley rats	↑ BBB rupture ↑ Tissue destruction ↑ Behavioral dysfunction	14 days	[209]
	Indirect/Focal	Male Sprague–Dawley rats	↑ Seizure susceptibility	6 months	[210, 211]

#### 3.1.2 FPI Model

The FPI model causes damage to the intact dura mater by creating a fluid pressure pulse through a pendulum striking the piston of a fluid reservoir. A craniotomy can be performed centrally, around the midline, or laterally, over the parietal bone, between bregma and lambda, to expose the dura mater [214, 215]. The percussion causes a temporary displacement and deformation of brain tissue, with the extent of the injury being influenced by the strength of the pressure pulse [141, 216]. This model successfully replicates the pathology of TBI in humans, even without skull fracture. It also displays key characteristics observed in clinical cases of TBI, including intracranial hemorrhage, brain swelling, and progressive disruption of gray matter [217, 218, 219].

In the development of the midline FPI model of brain damage, rabbits and cats were initially utilized [220]. Ultimately, the experiment was tailored to specifically target and affect only one hemisphere in rats and mice, following modifications made for the rat [221]. Using a small hole in the parietal bone, a controlled fluid pulse is directed toward the intact dural surface of the brain to cause diffuse brain injury, as depicted in Figure 2. The brain experiences vascular and axonal damage caused by the fluid percussion or lateral fluid percussion (LFP), regardless of the level of calcification in the bones and thickness of the skull [141, 216]. Based on the trajectory of the percussion waves and the location of the craniotomy, the FPI model can lead to complex patterns of brain malformation. Extensive subcortical neuronal damage is observed in rats with LFP injury, along with focal cortical contusion. This includes injury in the thalamus and hippocampus [140]. While this process starts soon after the collision and leads to neuronal death within 12 h, it is interesting to note that other brain areas do not show any significant damage even after 7 days. In terms of the craniotomy position concerning the sagittal suture, this model can be classified into three categories: midline (directly on the sagittal suture), parasagittal (located to the side of the midline, within 3.5 mm), and lateral models (positioned further away from the midline, more than 3.5 mm) [222, 223, 224].

Undoubtedly, the FPI model necessitates careful and thorough preparation of the animals, along with a high level of operational expertise, to accurately replicate pressure and the subsequent damage [28, 219]. Modifying the pendulum height can alter the force of the fluid pressure pulse delivered through the saline reservoir, thereby impacting the dura over the brain. In order to achieve more reliable and consistent brain compression patterns, researchers have made modifications to the damaged device. Instead of using a pendulum, they have incorporated a high‐pressure pump and a saline reservoir [119, 225, 226].

According to the FPI model, a human TBI can occur without a skull fracture and can lead to edema, gray matter injury, and hemorrhage [227]. As observed in concussive sports injuries, the primary types of injury caused by fluid pressure impulses are shearing/stretching of tissue, subdural hematoma, contusion, and bleeding [140]. The injury typically occurs after cortical contusions on the same side of the brain, along with damage to the external and internal capsules and the corpus callosum. These findings are consistent with traumas observed in humans [28]. As discussed in a study by Ma et al., neurobehavioral disorders observed in patients with TBI include reflex suppression, cognitive impairment, and vestibulomotor dysfunction. These symptoms are often a result of specific areas of neuronal loss in the brain [228].

In the study involving *Rattus norvegicus* (Rn) (GSE64986) conducted by Meng et al., adult male Sprague–Dawley rats were subjected to injury using the FPI method. Following the injury, they conducted RNA‐Seq analysis on the hippocampus and leukocytes collected 7 days later. The study outcomes revealed that TBI had a profound effect on the alternative splicing of genes related to a wide range of functions. These functions encompass neurons, complement and coagulation, transcription factors, blood pressure regulation, inflammation, mitochondria, leptin signaling, insulin signaling, and extracellular matrix genes. In addition, the study discovered a significant alteration in DNA methylation patterns in these genes, which were observed in both the hippocampus and leukocytes [229]. Thus, the FPI model can be seen as a viable TBI model for investigating the symptoms linked to TBI.

Nevertheless, the LFP model can also induce post‐traumatic epilepsy (PTE) in animals, but the incidence and severity can vary depending on factors such as the severity of the injury, the animal species, and the time course of the injury [230, 231]. Indeed, PTE is a debilitating condition characterized by recurrent seizures that develop as a consequence of brain injury and is considered one of the most devastating long‐term complications of TBI [232, 233]. In an early study, D'Ambrosio and colleagues affirmed that a single episode of severe FPI in rats can lead to PTE, as evident by spontaneous chronic seizures, originating from the injured neocortex, which progressively worsen and spread over time. Additionally, FPI‐induced epileptic rats displayed behavioral changes, such as pauses, facial automatisms, and myoclonus, during epileptiform events, thereby validating the utility of the FPI model as a valuable tool for studying the underlying mechanisms of spontaneous epileptogenesis [234]. Similarly, another study reported a PTE incidence of 43%–50% following FPI, with seizures typically developing within 7 weeks to 1 year post‐injury. The average seizure frequency remained consistent at 0.3 ± 0.2 seizures per day, lasting approximately 113 ± 46 s [235]. The endogenous cannabinoid system has also been associated with inducing FPI‐mediated PTE in rats. Results affirmed that the endocannabinoid system ECS demonstrated a biphasic response following brain injury. In the PTE group, the expression of the CB1 receptor and 2‐arachidonoylglycerol (2‐AG) was significantly elevated compared to the non‐PTE group 12 months after TBI [187]. Interestingly, it was found that FPI‐mediated intestinal permeability disruption and endotoxemia can augment PTE [188]. Furthermore, Meadel‐Matus and the team observed that LFP injury augments the severity of neuromotor impairments and PTE due to the modulation of the gut microbiome profile [189]. Succinctly, the ability of an FPI model to induce PTE is an important consideration when studying the long‐term consequences of TBI and evaluating potential treatments for this condition. However, further research is needed to better understand the factors that contribute to PTE development in animal models and to develop more reliable and predictive models for this condition.

#### 3.1.3 CCI Model

The CCI model is a percussion model that incorporates principles found in various impact models, such as the impact accelerator, weight drop, and FPI models [219, 236]. A comprehensive understanding of the factors that contribute to focal brain injury, such as injuries caused by boxing, ballistic injury, and contusion, can be obtained through laboratory experiments using animal models of CCI [228]. The model demonstrates a capacity for producing precise tissue distortion and carefully controlled variables such as velocity, impact time, and depth. Additionally, it effectively mitigates the potential for rebound injury, as noted in previous studies [237, 238, 239]. The CCI model is believed to offer a more comprehensive understanding of the biochemical, molecular, and cellular mechanisms underlying brain damage. This is due to its ability to induce more extensive brain tissue destruction and its precise control over mechanical parameters [143, 240, 241].

Cortical impact damage occurs when a rigid object transfers mechanical energy to the undamaged dura through air pressure [146]. The concept was initially created using ferrets [242] as test animals before being applied to rats or mice [243], and swine [244]. An experiment was conducted where a rod was propelled at high speed toward the brain through a surgically exposed dura. The impact's depth was carefully controlled using computer‐guided software. The size and geometry of the rod can be modified to accommodate various species, and its control mechanism is either electromagnetic or pneumatic, as described by Shultz et al. in 2017 [144]. Upon impact, the rod swiftly makes contact with the vulnerable brain tissue, resulting in the disruption of blood vessels and subsequent damage to the BBB, cellular structures, cortical tissue, and internal bleeding within the brain [245]. The severity of tissue damage and the speed of the rod can differ [246]. As an example, brain injury was induced in rats by Yu et al. at different depths for different levels of severity. The depths were 0.5 mm for mTBI, 1.0 mm for moderate TBI, and 2.0 mm for severe TBI. The velocity used was a constant 6.0 m/s [247]. In a different study, researchers induced varying levels of injury in mice by using different depths of 1.5, 2.0, and 2.5 mm [248]. Wang et al., on the other hand, introduced mild, moderate, and severe injuries in mice at depths of 0.2, 1.0, and 1.2 mm, with a velocity of 3.5 m/s, respectively [249].

It is worth noting that Meaney et al. conducted a study on the biomechanics of mTBI. They used a modified CCI model, adjusting the mechanical parameters and altering the material and size of the impactor tip. The modified CCI model utilizes a comparable methodology and equipment to the previously mentioned CCI model but incorporates a considerably reduced impact velocity. This modified model exhibits subcortical axonal injury, even in the absence of visible lesions or hemorrhage, which is a noteworthy characteristic. An interesting finding emphasized in this study is the lack of cortical lesions observed in both the sham and modified CCI brain images [147].

Indeed, CCI is widely recognized as a contributing factor to cognitive impairments and functional abnormalities. Several studies conducted by scientists using mice and rats have aimed to explore the significance of this model in comprehending the effects of such damage [214, 248, 250]. Studies conducted on rats and mice have observed a noteworthy connection between the impact‐induced deformation's velocity and depth, and the resulting cognitive deficits. This correlation was assessed using the Morris water maze test [214, 250]. Studies have indicated that these cognitive impairments can persist for a year and could be associated with a gradual reduction in blood flow to the brain and the development of brain shrinkage after CCI [191, 251].

In addition, CCI was found to be a contributing factor in the development of altered emotional behavior in mice. This was determined through various tests, including the elevated plus maze, prepulse inhibition of acoustic startle, and forced swim test [251]. In addition, scientists have pointed out that the swine CCI model shows consistent injuries with pathophysiological characteristics similar to those seen in human TBI. This makes it a promising method for translating data from in vivo studies to clinical practice [147, 252]. However, in a preliminary investigation conducted by Zhong et al., they used a CCI model to induce TBI in male C57BL/6 mice that were 12 weeks old. This study identified a total of 64,530 transcripts, with 27,457 being classified as mRNA and 37,073 as long non‐coding RNA. A comprehensive KEGG pathway analysis was conducted upon analyzing the differential expression in the RNA‐Seq data. This analysis revealed the involvement of a total of 234 pathways. Among the most enriched pathways were the MAPK signaling pathway, NF‐κB signaling pathway, ECM–receptor interaction, cytokine–cytokine receptor interaction, chemokine signaling pathway, phagosome, PI3K‐Akt signaling pathway, cell adhesion, osteoclast differentiation, complement, and coagulation cascades [253]. Interestingly, similarly to FPI, the CCI model has also been shown to elevate seizure susceptibility after TBI, as evidenced by diverse experimental evidence [193, 194, 195, 197, 254, 255, 256]. In a study by Eslami et al., it was found that TBI animals exhibited a significantly lower number of pentylenetetrazol (PTZ) injections (7.1 ± 0.3 injections) required to induce generalized seizures compared to sham‐operated animals (13.1 ± 1.6 injections) [198].

While this model offers advantages due to its incorporation of precise automated control over various factors, including impactor diameter, velocity, depth, and dwell time of impact, further standardization is still necessary. The variation in impactor tip hardness, although providing flexibility, also underscores the need for additional efforts in standardizing the method. This would enhance the reliability and reproducibility of the model, ensuring consistent and accurate results, especially when exploring a range of injury outcomes in subsequent studies.

#### 3.1.4 Closed‐Head Impact Model of Engineered Rotational Acceleration Model

CHI models are considered highly relevant TBI models because a significant number of human TBIs occur as a result of impacts on intact skulls [200]. However, traditional CHI models frequently show considerable experimental variation, which may be attributed to the limited control over biomechanical inputs. Advancements in pre‐clinical TBI research have resulted in the creation of the Closed‐Head Impact Model of Engineered Rotational Acceleration, also known as CHIMERA. This model was specifically developed to meet the requirement for a simple and dependable rodent CHI model that effectively mimics the majority of TBI cases in humans [127]. The chimera model has been used to study the effects of mTBI on neural stem cell proliferation and differentiation [257, 258]. In order to induce CHI in animals, researchers have developed three different CHIMERA devices. These devices are specifically designed for use in mice, rats, and ferrets, and are used to simulate injuries that occur from impacts and the resulting high‐velocity movement of the head and upper torso [259]. A high‐pressure impact is administered to the mice CHIMERA model using a metal piston. This piston strikes the dorsal surface of the head, delivering well‐defined energy hits to a closed skull. The mice have unrestricted head mobility after the impact [127, 259, 260]. This distinctive characteristic allows CHIMERA to effectively combine biomechanical, behavioral, and neuropathological studies [259]. In contrast to other neurotrauma model systems, CHIMERA enables precise impacts with predetermined dynamic parameters on a closed cranium, while also facilitating the analysis of unrestricted head movement kinematics [127, 200, 259].

The groundbreaking work of Namjoshi et al. introduced a pioneering nonsurgical impact acceleration model of CHI, which has proven to be a reliable method for inducing DAI and white matter inflammation. The CHIMERA model of repeated TBI replicates various functional and pathological features seen in human TBI, demonstrating a consistent biomechanical response of the head. This characteristic makes CHIMERA highly suitable for conducting detailed research on the underlying mechanisms of TBI and the creation of drug development initiatives. It serves as a dependable framework for examining the impacts of repeated TBI and potential therapeutic approaches [127, 260].

In a recent study, Basir et al. created a new TBI model for mice. The animals were exposed to a single closed‐head impact using specific equipment, including a stainless steel piston and a 3D‐printed PLA interface. The findings presented strong evidence of acute neurological impairments, heightened neuroinflammation, microgliosis in the gray matter, abnormalities in microstructural vasculature, as well as elevated levels of plasma total tau and neurofilament light [200]. CHIMERA allows for the examination of the impacts of mild repetitive injuries, which are commonly observed in cases of TBI. This procedure does not require surgery, making it an effective method for scientific study. Although this method effectively tackles scaling issues in pre‐clinical animal models, scaling up to humans remains a significant challenge. Like other impact models, it is crucial to find a delicate equilibrium in the severity of the injury. This involves providing sufficient force for tissue damage caused by acceleration, while also minimizing compression injuries.

#### 3.1.5 ACHI Model

The awake closed‐headed Injury (ACHI) model utilizes an adapted controlled cortical impactor to induce closed‐headed injury, leading to noticeable behavioral impairments. This method eliminates the need for a craniotomy or anesthesia, as demonstrated in previous studies conducted on adult mice [152, 153]. The experimental setup utilized specialized equipment to ensure precise and controlled conditions. This included the use of cones for nostril ventilation, which were securely fastened with plastic hair clips. Additionally, custom 3D‐printed plastic helmets were employed to effectively distribute impact force and reduce the potential risk of skull fractures. The helmets were carefully secured in place using elastic bands and tape, and their positioning was meticulously based on anatomical landmarks. An altered cortical impact device, equipped with a rubber tip, administered precise impacts to the left parietal cortex of rats positioned on a foam platform below. The impact parameters were meticulously regulated to guarantee precision and minimize the risk of harm. Following each impact, participants were immediately [154, 155, 202, 261]. This method often leads to minor injuries that do not result in death, skull fractures, or brain hemorrhages, which makes it ideal for studying repetitive mTBI (r‐mTBI) [144, 155].

Based on the available evidence, it appears that r‐mTBI can result in a progressive accumulation of damage, manifesting as behavioral symptoms, neuropathological changes, and neurodegeneration. It has been noted that r‐mTBI often occurs in young individuals who participate in sports during a time when their brains are undergoing important changes in synaptic connections and myelin formation. This specific group is especially susceptible to the lasting consequences of r‐mTBI [144].

A protocol has been developed by researchers to consistently and safely induce mild ACHI in rats, without causing any long‐lasting pain or mortality [154, 155, 202]. In addition, a new rapid neurological assessment protocol (NAP) has been developed, which can be completed within just 1 min after each impact. This ACHI model seeks to investigate the limited data available regarding the impact of repeated mTBI on the developing brain, particularly in juveniles who may experience longer lasting behavioral consequences. In addition, the ACHI model has the potential to provide valuable insights into the long‐term effects of early‐life r‐mTBI on brain neuropathology. It can also help identify specific periods of vulnerability to r‐mTBI across the lifespan [154]. Another study examined the synaptic function in the hippocampus of juvenile rats who experienced r‐mTBI using the ACHI model. Using a tissue slicer, hippocampal slices were created to examine the bidirectional synaptic plasticity at either 1 or 7 days after r‐mTBI. In general, the ACHI model provides researchers with a scientifically sound method to investigate alterations in synaptic plasticity after mTBI and r‐mTBI [202]. Further, in the study conducted on juvenile rats, it has been found that repeated impacts over 2 days impaired spatial memory. Interestingly, these impacts did not cause any structural deformities or motor impairments. This study confirms the reliability and efficiency of the r‐mTBI model in assessing TBI [155].

#### 3.1.6 Lateral Impact Model

mTBI models are essential in understanding the pathophysiology and consequences of brain injuries. The lateral impact model is a widely used and well‐established model of mTBI in preclinical settings. This model typically involves delivering a controlled impact to the lateral aspect of the animal's head, simulating the rotational and linear forces, thereby mimicking the common mechanism of injury in many real‐world scenarios, such as sports‐related concussions or motor vehicle accidents. The lateral impact model has been used to study various aspects of mTBI, including behavioral, cognitive, and histological changes [53, 258, 262]. The lateral impact model can also be widely useful for investigating the effects of unilateral injuries and assessing the potential for contralateral damage. Like the ACHI and modified weight drop models, the lateral impact model aimed to replicate clinically relevant biomechanics and pathophysiology of mTBI [154, 174].

Repetitive lateral impact via acceleration/deceleration and rotational forces also produces working memory deficits, loss of consciousness, motor and balance deficits, following augmented anxiety‐like behavior [263, 264, 265, 266]. Accumulating evidence affirmed that the lateral impact via acceleration/deceleration and/or rotational forces significantly augments acute behavioral impairments along with a deficiency in tasks related to emotional functioning [267, 268]. Lateral impact‐induced r‐mTBI has also been shown to impair the glymphatic system in limbic and cortical structures, potentially contributing to the development of post‐concussive symptomology during adolescence [257]. Interestingly, it was also observed that r‐mTBI via lateral acceleration/deceleration and rotational forces exhibited a sex‐dependent response [269]. Male rats subjected to TBI exhibited short‐term working memory deficits, while female rats displayed increased depressive‐like behavior. Additionally, sex‐specific alterations in the brain mRNA expression of GFAP, myelin basic protein, and tau protein were also observed following injury [262]. Furthermore, r‐mTBI induced by the lateral acceleration/deceleration model altered nociception and reduced anxiety levels in rats. Additionally, the results also observed a loss of orexin cell bodies and a decrease in hypothalamic projections to the ventrolateral nucleus of the periaqueductal gray (PAG), thus affirming that r‐mTBI via lateral impact can have significant effects on pain processing, emotional behavior, and neurotransmitter systems [270].

### 3.2 Animal Model of TBI Without Direct Head Impact

#### 3.2.1 Blast‐Induced TBI (bTBI)

Explosive forces can result in brain injuries even without a direct impact on the head. There are various factors that play a role in causing these injuries. These include shock waves produced by the explosion, objects propelled by the explosive device, additional damage to the brain, and involuntary movements of the head caused by conditions such as hemorrhagic shock, burns, and respiratory damage [271]. While the precise mechanisms of shock wave‐induced brain damage remain unclear, proposed mechanisms include the elevation of internal pressure during an explosion, leading to increased blood supply to the brain and subsequent neurological problems. Additionally, a shock‐bubble effect may occur in brain tissue due to an acoustic impedance mismatch caused by shock waves [272], and mitochondrial dysfunction may play a crucial role [273]. Notably, a shock wave is generated when a membrane separating two chambers with different air pressures ruptures, exposing animals inside the lower pressure compartment to a brief and intense shock wave of high pressure [274]. This model accurately replicates the significant morbidity associated with bTBI, which is especially common among military personnel. There are multiple factors that can contribute to the occurrence of bTBIs, such as shockwaves from explosions, direct projectile injuries, acceleration and deceleration injuries, as well as systemic variables like burns, hemorrhage, or respiratory arrest [275, 276, 277].

In rodent models, a frequently used approach to investigate the mechanism of bTBI is to expose the animals to open‐field blast explosions using TNT or similar explosives. This approach seeks to recreate blast exposures that occur in real‐life situations. It includes primary and reflected waveforms, as well as blast wind [278]. Shock tubes have long been utilized as a valuable tool in studying blast injuries in animals. These tubes consist of a cylindrical cast‐iron tube, measuring 400 mm wide, with a cone‐shaped tip and blast explosives made of pentaerythritol tetranitrate (PETN). In this experiment, the rats were carefully placed 1 m away from the detonation source and securely held in place using metallic nets. This was done to minimize any potential secondary effects caused by the blast [279, 280]. Various factors associated with shock tube experimentation can influence behavioral outcomes. These factors include the animal's positioning, orientation, use of head restraint, the end conditions of the blast tube (open or closed), and the length of the tube. The parameters mentioned in the study have a crucial influence on how animals respond to blast‐induced stimuli and can affect the behavioral outcomes observed in experimental studies [281, 282, 283].

Open‐field explosions subject rodents to the various effects of the blast, including primary, secondary, tertiary, and quaternary effects. However, the findings derived from animal models utilizing shock tubes can frequently be accompanied by uncertainties. This phenomenon is mainly caused by the intricate sequence of shock waves produced by shock tubes. These shock waves have a significant impact on experimental observations and can potentially lead to inaccurate results in studies focused on understanding the mechanisms behind bTBI [284]. As a result, these models frequently lead to increased mortality rates and pose difficulties in managing clinical outcomes in the animals. In addition, when conducting open‐field experiments, a greater amount of explosives is required to achieve the desired blast pressures compared to replicating similar pressures in shock tubes [278].

Several abnormalities arise from mild bTBI, causing modifications in the cortex and hippocampus, disruptions in the BBB, oxidative stress, and attracting the attention of numerous researchers who utilize this model. In a study conducted by Ratliff, the research centered around examining the effects of mild bTBI on the dendritic architecture of the cortex and hippocampus in mice. The results indicated a decrease in the intricacy of the dendritic arbor in both the parietal cortex and hippocampus. The observed morphological change indicates a possible connection to the cognitive and memory impairments that have been found in individuals with blast injuries, including humans. This study highlights the importance of gaining knowledge about changes in cell structure to better understand the cognitive effects of mild bTBI [285]. An analysis was also performed on rats that had experienced blast injuries, revealing alterations in the expression of gene families associated with inflammation, cell death, and neurotransmitters in the hippocampus. It was also observed that genes related to neurogenesis and synaptic transmissions were downregulated [210, 286].

In addition, Rubovitch's study uncovers long‐lasting cognitive and emotional behavioral abnormalities that occur after exposure to low‐level blasts in a realistic environment. The study reveals the correlation between changes in the BBB and the expression of certain biomarkers associated with oxidative stress (MnSOD2) and endovascular remodeling (CXCR3). These changes could potentially be signs of chronic neurologic complications, even if there are no obvious physical alterations in the brain or elsewhere. It is worth mentioning that there were no noticeable immediate alterations in neurological assessments. Based on the findings, the study indicates that blast‐induced mTBI may be considered a separate neurological condition. This calls for additional in‐depth research to better understand its underlying mechanisms [156].

#### 3.2.2 Acceleration–Deceleration–Induced TBI Model

Without any visible signs of head trauma or skull contact, the repeated and forceful shaking of the head can cause brain damage. This is because the brain moves unpredictably inside the skull during such movements [208, 287, 288]. Behavioral regression is a common short‐term symptom of accelerated–decelerated–induced brain injury. However, the long‐term implications of this type of injury can be more serious, leading to mental health issues and persistent neuropsychiatric complaints [276, 289, 290]. When a child's torso experiences vigorous shaking, it is referred to as “shaken baby syndrome” (SBS). Undesirably, this condition is frequently disregarded because there is no visible damage to the scalp [291]. Infants and young children have underdeveloped neck muscles that are not strong enough to control the rotation or acceleration of the head [287].

Although SBS is typically seen in infants under 2 years old, there have been documented cases in older children, with some as old as 7 years [292]. When the brain collides with internal bony structures in the skull, it can lead to various complications such as subarachnoid, subdural, and retinal hemorrhages, as well as encephalopathy. These complications can have a significant impact on motor and cognitive function, as well as vision [287]. During experiments with mouse models of SBS, anesthetized pups were gently shaken for 15 s at a rate of 900 cycles per minute (CPM) on a horizontally rotating shaker. Brain hemorrhaging was observed on postnatal day 13 (PN‐13). Between PN‐15 and PN‐31, there was a noticeable rise in the occurrence of cysts. After being subjected to shaking, animals showed specific damage in the white matter of their brain hemispheres. By PN‐31, it became clear that there was a significant reduction in the size of the white matter in the hemispheres [276, 293].

Understanding the impact of applied forces on head acceleration–deceleration is crucial in determining the extent of brain damage. Adults can sometimes experience a similar injury when their head moves unexpectedly in a forward and backward motion during car accidents. This can happen when the muscles in the neck are unable to handle the strong forces involved, resulting in a whiplash injury [288]. In a study conducted by Krave et al., it was found that rotational acceleration trauma in the sagittal plane of the head and neck led to widespread brain injury. Interestingly, the study also revealed that flexion caused more extensive damage than extension, even when the applied loads were identical [294]. In a similar vein, Wang et al. conducted a study on the effects of rotational acceleration‐deceleration on developing mice. Their findings indicated a potential mechanism that could contribute to secondary brain injury: hypoxia/ischemia. This research seeks to gain a deeper understanding of the physiological and behavioral effects of repetitive head injuries and the mechanisms that drive them. This mouse model's findings may offer valuable insights into the effects of rotational acceleration‐deceleration on brain health, especially in relation to pediatric head trauma [208].

Various acceleration devices have been developed for rodents, but unfortunately, many of them tend to cause more serious injuries, often resulting in meningeal bleeding [280]. Davidson and Rilsling developed a device that can generate controlled sagittal acceleration to the rat's head, resulting in DAI. In this model, the skull of a sedated adult rat was firmly attached to a rotating bar. When trauma is induced, a striker hits the bar, causing both the bar and the animal's head to rotate backward. During the acceleration phase, which lasts for 0.4 milliseconds, there is a subsequent rotation at a consistent speed, and a gradual deceleration occurs upon contact with a padded stop. The maximum allowable change in head angle is limited to 30°. Through manipulation of the air pressure in the rifle that propels the striker, a wide range of rotational accelerations, from 0.3 to 2.1 Mrad/s2, can be achieved [295]. This rotational trauma model is designed to generate graded DAI while minimizing the occurrence of contusion injuries. In addition, the TBI model designed can be replicated reliably and leads to a minimal occurrence of other forms of TBIs. As a result, it is highly valuable for studying injury biomechanics, diagnostic methods, and treatment strategies related to DAI.

#### 3.2.3 Penetrating Ballistic‐Like Brain Injury Model

Penetrating ballistic‐like brain injury (PBBI) occurs when high‐energy projectiles create a temporary cavity in the brain that is significantly larger than the projectile itself [30]. The result in PBBI is intricately linked to the trajectory of the projectile and the transfer of energy. In previous studies, researchers examined the effects of PBBI on cats by simulating gunshot wounds using a penetrating missile model [162, 296, 297]. A rat model of PBBI has been established, showing cognitive impairment and significant brain damage, such as damage to white and gray matter, swelling, and neuroinflammation [296, 297]. Therapeutic evaluations in this model have involved the use of dextromethorphan and human amnion‐derived multipotent progenitor cells [298, 299]. Accumulating evidence revealed that the PBBI model also has a wider susceptibility to augment PTE and epileptogenesis, similar to that of FPI and CCI models, and can be used as a preclinical model for underlying pathogenesis and evaluating potential therapeutic interventions for PTE [210, 211].

Recent advancements involve the introduction of novel rodent models for PBBI. One such model utilizes a modified air rifle to induce cavity formation, white matter degeneration, hemorrhage, and gliosis, without causing fatality [209, 300]. Yet another PBBI rat model has been developed to investigate the rapid and short‐term effects on intracranial pressure, BBB permeability, brain edema formation, as well as motor and cognitive impairments. There are similarities between the pathophysiological features of PBBI and other brain trauma models. However, PBBI stands out due to the significant intracerebral hemorrhage caused by the penetrating nature of the injury and the formation of a temporary cavity [298, 300, 301]. The PBBI model is highly effective in capturing the different temporal aspects of ballistic brain injury, which greatly enhances its value for conducting mechanistic studies and developing therapeutic interventions for moderate‐to‐severe brain trauma.

### 3.3 Miscellaneous Animal Models of TBI

mTBI is frequently seen in contact sports such as boxing, basketball, football, and rugby, as well as in cases of domestic violence. Research indicates that multiple concussions can lead to changes in behavior and pathological alterations. Indeed, various models of mTBI, including CCI, weight drop model FPI, and bTBI, have been established to study these effects [29, 184, 302, 303, 304, 305]. However, mTBI rarely occurs in isolation but is often accompanied by other injuries, such as fractures, burns, or internal bleeding, thereby exacerbating numerous complications [306, 307]. Polytrauma, a common consequence, especially in emergency settings, presents significant challenges including increased risk of infection, organ failure, and long‐term disability [308]. Over the past decade, rodent models of multi‐trauma/polytrauma involving TBI have been developed, providing valuable insights into how multiple injuries interact to influence systemic and central inflammation, as well as the structural and functional outcomes of TBI [306, 307]. Early polytrauma models utilize closed tibial fracture following an FPI‐induced TBI model to mimic multiple traumas, but unstabilized tibial fracture limited the ability to assess locomotor function between groups. However, these experiments revealed significantly elevated circulating levels of IL‐6 and IL‐10 within the first 24–48 h post‐injury in animals with combined injuries compared to those with isolated TBI [309, 310]. Similarly, Probst and team utilized a polytrauma model incorporating weight‐drop TBI, femoral fracture, and immediate hemorrhagic shock to mimic systematic inflammatory responses after polytrauma [311]. Similarly, Mirzayan et al. standardized the TBI model of polytrauma following a volume‐controlled hemorrhagic shock after CCI‐induced TBI and guillotine‐mediated femur fracture [312].

Furthermore, within a brief timeframe, mTBI can lead to DAI and chronic neuroinflammation [313]. Yang and colleagues observed significantly higher levels of IL‐1β, TNF‐α, and IL‐6 in the brain tissue of mice with combined TBI and bone fracture compared to those with TBI alone at 4 days post‐injury [314]. Similarly, tibial fracture following TBI augments neuroinflammatory responses at 24 h and 35 days after injury, along with significant disruption in BBB integrity [315]. Furthermore, polytrauma models with fractures elevate microglia and astrocyte activation following increased mRNA levels of several pan‐macrophage and pro‐inflammatory microglial/macrophage markers [316]. Studies by Weckbach et al. revealed that a combination of fracture, contralateral soft tissue injury, chest trauma, and closed head injury significantly augments the systemic and intrapulmonary release of cytokines, chemokines, and complement anaphylatoxins. Furthermore, the level of BAL protein and lung myeloperoxidase was also found elevated in polytraumatized rats affirming apoptosis [317, 318]. Notably, a polytraumatic model involving fracture, muscle crush, and TBI not only augments neuroinflammatory responses and behavioral impairment but has also been found to modulate the persistence of mechanical nociception [319, 320]. Nevertheless, the concomitant TBI and long bone fracture model leads to the increased size and bone volume of callus due to neurological insult [321]. Bardy and team developed a novel model that accurately mimics TBI‐induced neurological heterotopic ossification involving femoral fracture, TBI, and muscle crush injury. Results affirmed more prevalent ectopic bone formation at 6 weeks post‐injury with higher ectopic bone severity scores, thereby offering a valuable platform for investigating the underlying mechanisms of TBI‐associated long‐term complications [322].

Apart from polytrauma, TBI followed by infection is also a serious condition that exacerbates brain damage and leads to long‐term neurological impairment. Recently, Shad et al. explored the influence of *Klebsiella pneumoniae* infection on acute TBI outcomes. Results suggest that TBI following infection up‐regulates the gene expression of several neuroinflammatory genes, leading to substantial damage to cortical and hippocampal brain tissue. Despite profound acute effects, the findings indicate that this particular infection had a limited impact on secondary injury processes within the brain following TBI [323]. Similarly, Baker and team affirmed that pre‐existing *Toxoplasma gondii* infection augments brain damage during TBI pathogenesis by modulating glutamatergic, neurotoxic, and oxidative stress markers, along with significant elevation in the expression of neuroinflammatory mediators [324]. Nevertheless, hospitalized TBI patients are more prone to infection [325, 326]. Multiple models have been established to replicate the septic state following TBI, frequently employing lipopolysaccharides (LPS), administered either intraperitoneally or intravenously, to modulate the peripheral immune system and facilitate inflammatory responses [327]. In a study by Sharma and team, 4 days post‐exposure to LPS in TBI mice significantly augments glial activation and expression of inflammatory cytokines [328]. Furthermore, it was also found that LPS infection following TBI also enhances the incidence of spontaneous seizures via a subtle increase in ipsilateral hippocampal tissue loss [194].

Intimate partner violence (IPV) can also lead to TBI, often through mechanisms like concussion or strangulation. Acute IPV patients with concussion and strangulation have been found to exhibit more severe brain injury biomarkers and symptoms. Recently, Sun et al. developed a novel rat model of non‐fatal strangulation, and the procedure involves placing a band around the trachea and suspending a 680‐g weight from it for 90 s while monitoring blood oxygen saturation (SpO2) with a pulse oximeter attached to the hind paw. The results affirmed that the novel model increases circulating NFL and GFAP levels, augments behavioral impairments, and promotes neuroinflammation [329]. In summary, these novel animal models incorporating additional insults, such as polytrauma, infection, and inflammation, offer a more comprehensive approach to studying TBI. These models can enhance clinical relevance by mimicking real‐world scenarios where patients often experience multiple injuries and complications. While these models present additional challenges, they can provide valuable insights into the complex interactions between TBI and other factors, leading to improved understanding of the disease and the development of more effective treatments.

## 4 Challenges and Future Perspectives

Using in vitro and animal models, as well as computational modeling of TBI, significant contributions have been made to our current understanding of post‐traumatic sequelae. Understanding how preclinical research translates into clinical applications remains a complex task. Various rodent models have been created to mimic the various injury mechanisms seen in human TBI. Animal models have played a crucial role in enhancing our comprehension of TBI and investigating possible therapies. Through replicating key aspects of TBI in controlled experimental settings, researchers can gain valuable insights into the underlying mechanisms, pathology, and recovery processes. Animal models serve as a valuable tool for testing new therapeutic methods, assessing their effectiveness, and establishing the appropriate safety measures, dosage, and timing of interventions before moving on to human clinical trials. Although animal models have their merits, it is crucial to acknowledge their limitations. Understanding the variations in anatomy, physiology, genetics, and behavior between animals and humans is crucial in determining how injuries are responded to and treatment outcomes are affected. It is crucial to thoroughly address ethical considerations. To address these limitations, it is important to combine findings from animal studies with clinical observations and translational research to ensure their applicability to the human population. By adopting an interdisciplinary approach, we can gain a deeper understanding of TBI and make significant strides in developing effective treatments for clinical practice. Additionally, it is important to focus on end‐point biomarkers, address comorbidities such as aging, and enhance mortality and morbidity outcomes. It would be beneficial to conduct studies for a longer period, possibly up to 1 year, in order to gain a more comprehensive understanding of the delayed progression of brain damage and metabolic changes in the brain. This extended timeframe could offer valuable insights into treatment options for patients who were unable to receive early treatment after a brain injury.

## 5 Conclusion

Animal models of TBI play a crucial role in advancing our understanding of TBI pathophysiology and in developing potential treatments. While no animal model can fully replicate the complexity of human TBI, they provide valuable insights into various aspects of the injury process. These models allow researchers to study the mechanisms underlying TBI, including primary injury caused by the initial trauma and secondary injury processes such as inflammation, oxidative stress, and neurodegeneration. By manipulating variables such as the severity, location, and mechanism of injury, researchers can simulate different aspects of human TBI and evaluate potential interventions. Moreover, animal models enable the preclinical testing of novel therapies, including pharmacological agents, stem cell therapies, and neuroprotective strategies. By assessing the efficacy and safety of these interventions in animals, researchers can identify promising candidates for translation to clinical trials.

To achieve a therapeutic breakthrough in TBI, it is important to take a comprehensive approach. This involves making advancements in clinical trial design, creating models that closely resemble human brain injury, refining existing models and functional tests, considering the impact of systemic insults, and using multimodality monitoring. Additionally, it is crucial to explore how factors such as age, sex, and species/strain can affect TBI outcomes, and to improve drug delivery systems for the brain. Furthermore, it is important to conduct thorough investigations into precise and delicate biomarkers, while also closely monitoring the levels and effects of targeted medications, in both animal models and clinical environments. However, it is also essential to acknowledge the limitations of animal models and recognize that findings may not always directly translate to human patients. Factors such as species differences, genetic variability, and the controlled laboratory environment can influence results and may limit the generalizability of findings to clinical settings. Therefore, while animal models of TBI are invaluable for advancing our understanding and treatment of this complex condition, their relevance in clinical settings depends on careful interpretation and validation of findings in human studies. Collaborative efforts between researchers, clinicians, and industry partners are essential for bridging the gap between preclinical research and clinical practice and ultimately improving outcomes for TBI patients.

## Author Contributions

**Payal Chauhan:** data curation, writing – original draft; **Nikita Yadav:** writing – original draft, writing – review and editing; **Karan Wadhwa:** data curation, writing – original draft, writing – review and editing; **Govind Singh, Abhilasha Ahlawat:** conceptualization, supervision; **Athanasios Alexiou, and Niraj Kumar Jha:** supervision, writing – review and editing; **Marios Papadakis:** funding, writing – review and editing; **Subbulakshmi Ganesan, Chakshu Walia, Gulshan Rathore, and Mosleh Mohammad Abomughaid:** revision. All the authors approved the final version of the paper.

## Ethics Statement

The authors have nothing to report.

## Consent

The authors have nothing to report.

## Conflicts of Interest

The authors declare no conflicts of interest.

## Data Availability

All available data are included in the manuscript.
